# Leprosy, Still Present in La Réunion

**DOI:** 10.3201/eid1801.111176

**Published:** 2012-01

**Authors:** Pascal Vilain, Sophie Larrieu, Guillaume Camuset, Nicolas Pouderoux, Anne Gerber, Gianandrea Borgherini, Sophie Fite, Laurent Filleul

**Affiliations:** Regional Office of French Institute for Public Health Surveillance of Indian Ocean, Saint-Denis, La Réunion (P. Vilain, S. Larrieu, L. Filleul);; Regional Hospital Center, Saint-Pierre, La Réunion (G. Camuset, G. Borgherini);; Regional Hospital Center, Saint-Denis (N. Pouderoux, A. Gerber);; Dermatology Office, Saint-André, La Réunion (S. Fite)

**Keywords:** bacteria, leprosy, autochthonous cases, La Réunion, Reunion Island, World Health Organization, surveillance

**To the Editor:** During recent decades, a considerable and consistent decrease in worldwide incidence of leprosy has been observed, mainly because of the recommendation to introduce multidrug therapy in 1981 (*1*) and the implementation of free therapy in 1994 (*2*) by the World Health Organization (WHO). The prevalence rate of the disease has been reduced globally by >90% since 1985 (*3*). Since 2000, WHO has recommended the implementation of a leprosy surveillance system in leprosy-endemic countries with indicators for screening, treatment, and monitoring of patients (*4*). From these indicators, WHO establishes an annual official report on the global status of the disease. According to official reports received from 141 countries, the global registered prevalence of leprosy was 211,903 cases in 2010 (*5*).

La Réunion is a French overseas department located in the Indian Ocean 700 km east of Madagascar. La Réunion’s health care system is similar to that of continental France. Although new cases of leprosy have been punctually reported by health professionals during the past few years, which suggests that the disease is still present, the situation in La Réunion is poorly documented because of the lack of a specific surveillance system. Thus, the goal of eliminating leprosy as a public health problem (i.e., prevalence <1/10,000) (*6*) cannot be assessed because the goal requires a good knowledge of the epidemiologic status of the disease. Furthermore, the risk of leprosy recrudescence linked to a relapse of patients with autochthonous cases or patients with leprosy migrating from neighboring leprosy-endemic countries, such as Madagascar, Comoros, and Mayotte (*5*), is present. In 2009, a total of 1,572 new cases of leprosy were detected in Madagascar, 319 in Comoros, and 37 in Mayotte (*5**,**7*). If one considers the geographic proximity and the many tourist exchanges between La Réunion and those neighboring islands, the risk of importation, although low, is constant.

In that context, Cire Indian Ocean (the Regional Office of French Institute for Public Health Surveillance), in collaboration with health professionals involved in diagnosis and treatment of the disease, has implemented a specific surveillance system for leprosy in La Réunion. The objectives are to guide potential preventive measures by determining incidence of leprosy, following the disease’s evolution, and characterizing the patients affected.

The surveillance system was based on the notification of every case by health professionals likely to diagnose and treat subjects according to the WHO case definition (*8*), i.e., clinicians, private or hospital dermatologists, and infectious disease specialists. The notification was realized through a standardized questionnaire that included sociodemographic, clinical, and microbiological data. Concurrently, the pathology laboratories were consulted to detect any nondeclared cases and to improve the completeness of data.

This surveillance was retrospective for 2005–2010, then prospective for 2011. In total, 17 patients responding to the case definition of leprosy and given a diagnosis during 2005–2010 were reported for an average population of 804,025 inhabitants in La Réunion (data from the National Institute of Statistics and Economics Studies). The mean annual incidence during this period was 3.4 cases/10^6^ inhabitants. The male:female sex ratio was 2.2, and the median age was 54 years (range 8–77 years). More than half the patients were born in La Réunion (n = 9); the other patients’ birthplaces were Comoros Islands (n = 4), Mayotte (n = 3), and Madagascar (n = 1). Among the patients born in La Réunion, 6 had never left the island, 3 had traveled but had always resided in La Réunion, and 6 patents resided in the same area of a city in the southwestern part of the island.

An active search for other cases in this area was performed by contacting all the health professionals likely to diagnose leprosies; 1 clinician reported a suspected case among his patients. That patient is currently being screened. Of the patients overall, 15 were screened by skin biopsy or smear from the ear. According to the microbiological classification, 14 patients had a multibacillary form (positive smear) and 1 patient had a paucibacillary form (negative smear). Clinical signs suggested multibacillary leprosy (>5 patches or lesions on the patient’s skin) for 15 patients and paucibacillary leprosy (1–5 patches or lesions on the skin) for 2 patients. The median time between diagnosis and treatment was 6 days (range 0–20 days). Four patients had a severe disability with a grade 2. Overall, 15 patients had lepromatous leprosy and 2 had tuberculoid leprosy.

Although elimination of leprosy was achieved in La Réunion, the implementation of a leprosy surveillance system enabled us to highlight an autochthonous circulation of *Mycobacterium leprae*, leading to a cluster of cases recently diagnosed in the southwestern part of the island. During the investigation of this cluster, it was noticed that most of the doctors were unaware of the existence of leprosy in La Réunion or of the disease’s clinical signs. Incidence of leprosy could therefore be largely underestimated because of this lack of knowledge, and actions to raise awareness among health care professionals will be established to improve the detection and rapid treatment of patients.
